# “It’s all about delivery”: researchers and health professionals’ views on the moral challenges of accessing neurobiological information in the context of psychosis

**DOI:** 10.1186/s12910-020-00551-w

**Published:** 2021-02-08

**Authors:** Paolo Corsico

**Affiliations:** Centre for Social Ethics and Policy, Department of Law, School of Social Sciences, The University of Manchester, Williamson Building, Oxford Road, Manchester, M13 9PL UK

**Keywords:** Psychosis, Neuroscience, Genomics, Information, Ethics, Qualitative, Narratives, Identity

## Abstract

**Background:**

The convergence of neuroscience, genomics, and data science holds promise to unveil the neurobiology of psychosis and to produce new ways of preventing, diagnosing, and treating psychotic illness. Yet, moral challenges arise in neurobiological research and in the clinical translation of research findings. This article investigates the views of relevant actors in mental health on the moral challenges of accessing neurobiological information in the context of psychosis.

**Methods:**

Semi-structured individual interviews with two groups: researchers employed in the National Health Service (NHS) or a university in England (*n* = 14), and mental health professionals employed in NHS mental health services (*n* = 14). This article compares results in the two groups (total *n* = *28*).

**Results:**

This article presents findings around three conceptual areas: (1) research ethics as mostly unproblematic, (2) psychosis, neurobiological information, and mental health care, and (3) identity, relationships, and the future. These areas are drawn from the themes and topics that emerged in the interviews across the two groups of participants. Researchers and health professionals provided similar accounts of the moral challenges of accessing—which includes acquisition, communication, and use of—neurobiological information in the context of psychosis. Acquiring neurobiological information was perceived as mostly unproblematic, provided ethical safeguards are put in place. Conversely, participants argued that substantive moral challenges arise from how neurobiological information is delivered—that is, communicated and used—in research and in clinical care. Neurobiological information was seen as a powerful tool in the process through which individuals define their identity and establish personal and clinical goals. The pervasiveness of this narrative tool may influence researchers and health professionals’ perception of ethical principles and moral obligations.

**Conclusions:**

This study suggests that the moral challenges that arise from accessing neurobiological information in the context of psychosis go beyond traditional research and clinical ethics concerns. Reflecting on how accessing neurobiological information can influence individual self-narratives will be vital to ensure the ethical translation of neuroscience and genomics into mental health.

**Trial registration:**

The study did not involve a health care intervention on human participants. It was retrospectively registered on 11 July 2018, registration number: researchregistry4255.

## Background

The convergence of clinical neurosciences, next-generation genomics, and data science is leading the way towards a deeper understanding of the neurobiology of mental illness [1, 2]. Psychotic disorders are among the most debilitating forms of mental illness [3, 4]. Two strains of research are currently shedding light on the neurobiology of psychosis. First, over the past decades neuroimaging has allowed researchers to identify neuro-cognitive correlates of psychosis [5]. Second, the expansion of molecular genomics and next-generation sequencing is playing a pivotal role in unveiling the basis of heritability of psychotic disorders as well as the molecular processes involved in disrupted neuro-cognitive development, which in turn leads to vulnerability to psychosis [6–8]. Several scholars further claim that the *convergence* of information availability and data science has the potential to transform mental health care via public health approaches and artificial intelligence [9–11].

Accessing neurobiological information in the context of psychosis generates several moral challenges. Not only must researchers deal with issues of mental capacity and research participants’ vulnerability [12]. Other theoretical and practical problems arise. First, given the difficulty of translating neurobiological findings into clinical applications, it might appear more difficult to justify neurobiological research in the first place. Joseph goes as far as advocating for a moratorium on schizophrenia genetic research [13]. From the opposite viewpoint, Insel has highlighted the need to rethink the very concept of schizophrenia while affirming the relevance of neurobiology in redefining diagnostic categories [14]. Second, neuroimaging and genetic research on psychotic illness generate ethical dilemmas. How should we manage incidental findings in psychiatric neuroimaging research [15, 16]? Is there a moral obligation to return the results of psychiatric genetic research to participants [17, 18]? Third, it is unclear what impact having access to neurobiological information may have on the identity of mental health patients and how neurobiological information could affect their family and social relationships [19]. Will having access to one’s neurobiological information be beneficial to the development of the self-narratives of those who experience psychosis? Will it be detrimental to their journey towards recovery?

This article does not tackle these issues with robust philosophical arguments. Nor does it support any strong normative claim. Rather, it provides a glimpse into the moral life of relevant actors in mental health. By ‘moral life’ I mean the ways in which different actors describe and frame the ethical challenges of their professional roles in the everyday practice of research and care. Historically, the convergence of neuroscience and genomics to tackle psychosis has been situated in an overly-polarised cultural milieu, which is very different from the one found in physical health. The fight between biological and psychosocial approaches to mental illness has been raging for decades and it is far from being resolved [20, 21]. Within this fight, biological psychiatrists often see the implementation of neuroimaging and genomics as a mandatory step towards the development of effective treatments and public health agendas, whilst psychosocial scholars tend to reject such framings on ethical and political grounds [9, 22]. Within this fight, the present article is an exercise of aetiological *neutrality*. It contends that by exploring the views of professionals with different backgrounds across the aetiological divide we might help to inform the ethical debate, at least by situating moral principles and obligations within the practical reasoning of the very individuals who should *enact* those principles and *fulfil* those obligations [23, 24]. Further, it builds upon the assumption that exploring such views may help bioethicists to redefine their arguments by considering real-world implications of principles and obligations [25].

This article presents findings from interviews that I conducted with two groups: researchers in mental health and mental health professionals. I sought to investigate how these groups understand, and respond to, the moral challenges of accessing neurobiological information in the context of psychosis. I investigated how researchers and health professionals conceptualise: (1) the moral challenges of conducting neurobiological research—that is, neuroimaging and genomic research—in the context of psychosis, and (2) the moral challenges of accessing neurobiological information within clinical interventions for psychosis.

Two terminological clarifications are needed. First, throughout the article I use the expression ‘in the context of psychosis’ to refer to research conducted with individuals with an established diagnosis as well as research that investigates neurobiology in prodromal or (healthy?) at-risk individuals [26]. I recognise that these two populations have different ethical and legal profiles [27]. However, the focus of this article is on the moral challenges of accessing neurobiological information *related to* psychosis—that is, information around genomic and brain correlates of psychosis, or information around risk status and illness susceptibility—regardless of the actual diagnosis of research participants and care recipients. This tension was made explicit to participants in this study, and an indication of which populations the different interview questions refer to can be found in the interview guides. I also clarify this in the results. However, a certain degree of ambiguity is maintained. I believe that this ambiguity may signal the pervasiveness of the ethical ramifications of biomedical innovation in psychiatry. Second, in this article I use the word ‘access’ as an *umbrella term* that comprises ‘acquisition’ and ‘delivery’ of neurobiological information. In turn, the term ‘delivery’ comprises ‘communication’ and ‘use’ of neurobiological information. Hence, the term ‘access’ comprises acquisition, communication, and use (as the last two terms constitute ‘delivery’). To specify further, in this article I use the word ‘acquisition’ only to describe actions performed by researchers or health care professionals and not by patients or service users: I do not consider direct-to-consumer applications. The words ‘access’ and ‘use’ describe actions performed by researchers/professionals *or* patients/service users. For example, a service user may access, or use, neurobiological information which (s)he did not acquire, if this information is communicated to or used with him/her. The words ‘delivery’ and ‘communication’—by definition—describe actions performed by professionals and patients/service users *together*.

## Methods

This article presents the first set of results of a larger research study entitled ELSI-NAPS: Ethical, Legal and Social Issues in Novel Neurobiological Approaches to Psychosis and Schizophrenia—a Qualitative Study. ELSI-NAPS also included focus groups with carers of a person suffering from a psychotic disorder. Focus group data are *not* discussed here.

### Data collection

One-time, semi-structured individual interviews were held with participants in two groups: researchers (group A) and mental health professionals (group B). Inclusion criteria for participants in group A were: (1) being a researcher in psychiatry, psychology, or mental health, with a research interest in psychosis or schizophrenia, employed in a National Health Service (NHS) facility or in a university in England; (2) good spoken English; (3) having a PhD or a clinical doctorate. Inclusion criteria for participants in group B were: (1) being a mental health professional with at least 1 year of work experience with psychotic populations, employed in an NHS community mental health service or inpatient unit; (2) good spoken English; (3) having an undergraduate degree. To account for variation of professional background, participants in group B included mental health nurses, social workers, clinical psychologists, and psychiatrists.

I used *purposive* sampling to identify potential participants [28, 29]. For group A, I identified researchers via websites of universities in England. For group B, I identified mental health professionals across community mental health services in Greater Manchester Mental Health NHS Foundation Trust. Potential participants were contacted via email and offered participation if they met inclusion criteria. All participants provided written informed consent and completed a demographic questionnaire prior to the interview. They received no incentive for their participation. Participants could have their travel expenses reimbursed if they wished so. Each interview lasted for approximately 45 min and took place either in the participant’s office, in a private meeting room at the participant’s workplace, or in a public meeting room.

I used two interview guides. I developed the interview guides in collaboration with my doctoral supervisors as identified in the ‘Acknowledgments’ section of this article. The interview guides were developed by identifying relevant topics in the academic literature on the ethical, legal, and social issues arising from neuroscientific and genomic approaches to psychosis, and by selecting those topics that were deemed appropriate for discussion with ELSI-NAPS participants. The interview guides were designed to direct discussion towards participants’ views as these related to their professional experience. Hence, the two guides focused on analogous ethical issues but diverged with regard to the context where ethical issues and moral dilemmas arise. For the researchers, the interview guide focused primarily on ethical issues arising in *clinical research* and then touched upon moral challenges in clinical practice. For the mental health professionals, the interview guide briefly referred to ethical issues in clinical research and then focused mostly on moral challenges in *clinical practice*. The interview guides are presented in Additional files 1 and 2.

### Data analysis

Data collection and data analysis were performed as an *iterative* process [30]. Data analysis began before data collection was completed. This allowed me start developing coding materials before data collection was completed and to inform the conduct of subsequent interviews. However, the interview guides remained the same during data collection to ensure consistency of topics covered. Interviews were audio recorded. Recordings were transcribed by a transcription service approved by the University of Manchester using an intelligent (clear) verbatim approach: all words in the recordings were transcribed but habitual hesitations were removed for ease of reading unless deemed essential to portray the conversation. Identifiable information was removed from the transcripts and pseudonyms were added to the transcripts in lieu of participant information. As per ELSI-NAPS data management plan (available from the Department of Law, The University of Manchester), the pseudo-anonymization key linking pseudonyms with participant information is stored separately from ELSI-NAPS research data as an encrypted and password-protected file at the University of Manchester. The pseudo-anonymization key will be erased after the end of my registration as a doctoral student at the University of Manchester. Without the pseudo-anonymization key, all ELSI-NAPS research data can be considered anonymised. Participant quotations included in this article do not contain identifiable information or pseudonyms. Transcripts were analysed in a stepped thematic analysis process [31]. After a first initial reading, codes were developed that captured the arguments articulated by participants. Codes (themes) were grouped in higher order categories and the categories were organised under different topics explored in the interviews. Two distinct coding structures and coding manuals for the two groups were inductively developed from the transcripts. In a second phase, two other researchers independently reviewed the coding structures and manuals against 5 of the 28 transcripts (3 transcripts for group A and 2 transcripts for group B) to ensure reliability. The coding structures and manuals were revised by incorporating reviewers’ comments, and consensus was reached. In a third phase, the transcripts were transferred to NVivo 11 software (QSR International) and the new coding structures and manuals were used to code all the transcripts. Some codes were eventually adjusted during this process. After all the transcripts had been coded I used the analysed data to compare results in the two groups and to write this article. The final thematic map which combines the two coding structures can be seen in Table 1. The final coding manuals are presented in Additional files 3 and 4.

**Table 1:**
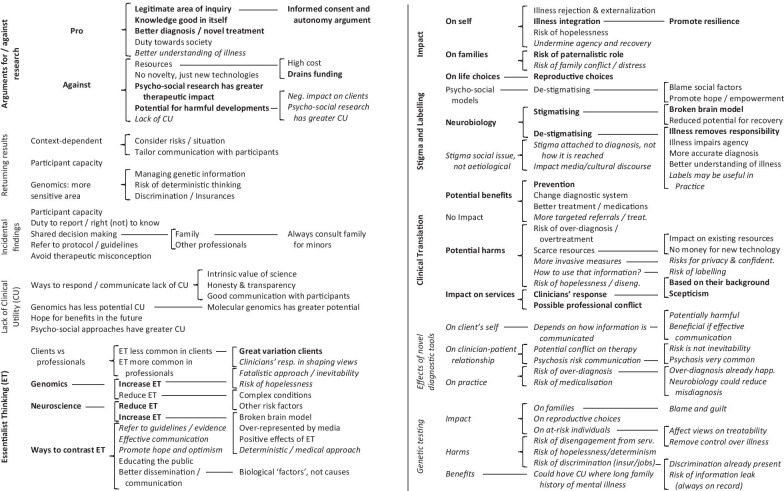
Thematic map: normal font = researchers; *italics* = *health professionals;* **bold = both**

*CU clinical utility, *ET essentialist thinking

## Results

14 researchers in group A and 14 mental health professionals in group B were recruited between November 2017 and July 2018 (total *n* = 28). Participant demographics can be seen in Table 2. Most researchers described their research field as either clinical psychology (*n* = 7) or psychiatry (*n* = 5). The majority of them stated that they had received some ethics education in the form of Good Clinical Practice (GCP) training, while half of them stated that they had received further ethics training. Most mental health professionals worked as either mental health nurses (*n* = 5) or social workers (*n* = 3). Half of them stated to have received GCP training, and only four said that they had received further ethics training. Table 1 presents the combined *thematic map*. Themes are organised into sub-categories which are then grouped into broader topics. Eight topics emerged from discussions with the researchers in group A: arguments for and against research, returning results, incidental findings, lack of clinical utility, essentialist thinking, impact, stigma and labelling, and clinical translation. Seven topics emerged from discussions with professionals in group B: arguments for and against research, essentialist thinking, impact, stigma and labelling, clinical translation, effects of novel diagnostic tools, and genetic testing.

**Table 2:** Participant demographics; *GCP good clinical practice training

	Age	Gender		Education	Ethics training
**Researchers** *n* = 14	[33–74] Mean = 45.3	M = 8 (57.2%) F = 6 (42.8%)	**Main research field** Clinical Psychology = 7 Psychiatry = 5 Education = 1 Psychosis = 1	PhD = 10 Clinical doctorate = 3 Both = 1	GCP = 12 (85.7%) Other = 7 (50%)
**Mental health professionals** *n* = 14	[37–64] Mean = 46.1	M = 6 (42.8%) F = 8 (57.2%)	**Occupation** Mental health nurse = 5 Social worker = 3 Psychiatrist = 2 Psychotherapist = 1 Counsellor = 1 Clinical psychologist = 1 Care coordinator = 1	Doctorate = 2 Postgrad. = 8 Undergrad. = 3 Not disclosed = 1	GCP = 7 (50%) Other = 4 (28.6%)

In this article I present results around three areas. These areas are drawn from the topics listed above whereby different topics are presented together—across the two groups of participants—because of their conceptual affinity. The section “research ethics as mostly unproblematic” presents results on: arguments for and against research in both groups, and returning results and incidental findings in group A. The section “psychosis, neurobiological information, and mental health care” presents results on: lack of clinical utility in group A, clinical translation in both groups, and effects of novel diagnostic tools in group B. Lastly, the section “identity, relationships, and the future” presents results on: essentialist thinking, stigma and labelling, and impact in both groups, and genetic testing in group B. Table 3 shows the relation between the three areas in the results and the topics presented in the thematic map in Table 1, as well as the participant group to which each topic refers.

**Table 3:** Topics discussed in each area of ‘Results’

Results area	Topics discussed
Research ethics as mostly unproblematic	**Arguments for/against research** Returning results Incidental findings
Psychosis, neurobiological information, and mental health care	Lack of clinical utility (CU) **Clinical translation** *Effects of novel diagnostic tools*
Identity, relationships, and the future	**Essentialist thinking (ET)** **Stigma and labelling** **Impact** *Genetic testing*

Groups: normal font = researchers; *italics* = *health professionals*; **bold = both**

### Research ethics as mostly unproblematic

Researchers and health professionals provided a number of arguments to justify conducting neurobiological research on psychosis. Despite the current lack of clinical applications, participants in both groups argued that neurobiology yields the potential to improve the understanding of psychosis, redefine diagnostic categories, and produce better treatments. The neurobiology of psychosis was generally recognised as a legitimate area of scientific inquiry regardless of participants’ professional background. Further, both researchers and professionals argued that individuals who suffer from a psychotic disorder should not be assumed to lack capacity to consent to research *only* because of their diagnosis. Individuals who are deemed to have capacity to take part in research should be treated as any other (healthy) individual with regard to providing informed consent. Two justifications were presented for this argument. The first justification focused on autonomy:

Again I suppose I keep returning to a kind of Kantian framework for this, if the patients are happy to take part and we want to do it, well who’s telling us we shouldn’t be doing what we all want to do, you know, where’s the big harm that nobody’s decided to take on?

researcher, psychiatry

According to this argument it would be *paternalistic* to take decisions regarding participation in neurobiological research on behalf of capacitous patients or service users. The second justification focused on justice and non-discrimination. It would be unfair to exclude capacitous individuals from neurobiological research because of their mental illness:

I would never make decisions on behalf of service users to say I really think this isn't good for you. I would weigh up some of those decisions with them personally but I'm of the opinion that I like to give people all the opportunities that might be [avail]able for them, and for them to make their own minds up.

health professional, clinical psychologist

On the other hand, many participants highlighted the fact that neuroscience and genomics are ‘costly’ enterprises. Neurobiological research risks shifting useful resources and draining funding away from psychosocial research which in turn—as some participants argued—has proved to have greater clinical utility. Some participants also stressed that research into the neurobiology of psychosis is not new and that the claimed novelty of neuroimaging and molecular genomics only lies in the use of more sophisticated technologies.

I asked researchers about returning results and disclosure of incidental findings. Researchers generally supported the idea that they have a *duty* to communicate aggregate results and that participants should, if they wish, be offered the opportunity to know the main findings. Interestingly, most researchers recognised that the same obligations apply to the disclosure of individual results, thus echoing recent debates which problematise the distinction of researchers’ obligations with regard to returning genomic results [18, 32]. Even though researchers recognised practical differences in returning aggregate or individual results, their main concern was not *whether* to disclose, but *how* to disclose such information—with reference to communication strategies—and what *type* of information is disclosed, as they highlighted significant differences between neuroscience and genomics in this regard. Researchers stressed the importance of tailoring communication with participants to the capacity, age, and clinical status of the individual, and the duty not to expose participants to potential risks related to disclosure of aggregate / individual findings:

I suppose if you’re having some investigations, then you have a duty to give the results, don’t you. So, I think that that’s important. I suppose it depends what the message is and how you deliver it and what’s taken away, and then the person’s understanding of what that means.

researcher, clinical psychology

Further, the presence of psychotic illness was perceived as a reason to develop appropriate communication strategies and to evaluate carefully the risk–benefit ratio of results communication. Again, a diagnosis of a psychotic disorder was *not* perceived as a valid reason to withhold information from research participants. At the same time, several researchers argued that genomics represents a more sensitive area compared to neuroscience, mainly because of the cultural discourse surrounding genomic information:

I suppose there’s an extra degree of problem often with genomics, because it’s so emotive, and people often seem to feel that genes are destiny, in a way that environment or kind of mediating processes aren’t, that’s of course less true in some ways. But because of that it’s very easy to tell people something that would make them feel doomed, and so I think there are special difficulties that arise from the difference in degree to which that can happen.

researcher, psychiatry

Despite the fact that recent literature questions the distinction between intended and incidental research findings [33], researchers in this study still recognised specific obligations when relevant ‘incidental’ or ‘unsolicited’ findings emerge from their research. Yet, managing incidental findings was perceived as not particularly problematic. Most researchers recognised that they have a moral obligation to report relevant, clinically-actionable incidental findings, and that research participants have a right (not) to know them. Researchers argued that the most reasonable way to manage incidental findings is to establish an appropriate course of action before the research takes place. The possibility that the research might generate incidental findings should be explained in participant information sheets. Participants should be informed of this possibility and given the opportunity to express their disclosure preferences. Procedures to deal with incidental findings should be described in the research protocol, and be subject to REC/IRB scrutiny. Such procedures should focus on shared decision-making among different actors and include the research participant, the family (if this was the participant’s preference), and other professionals:

Ideally you’ve already got a protocol in place about how you manage it, you have to be ready for it, […] Often it’s about having a clear protocol around how you signpost people, and ensure they get the support they need, now it’s the same with, you know, brain imaging, if you find anything you are expecting is problematic you should have a kind of plan of how you signpost or direct those people to kind of the support or additional checks they need, to help them.

researcher, clinical psychology

Interestingly, most researchers argued that where (1) a pre-determined course of action was clearly described in the research protocol, (2) this course of action was based on established guidelines, and (3) the preferences of a capacitous individual were respected, then disclosure of incidental findings would not be a particularly challenging moral dilemma. The interplay among research protocol, REC / IRB scrutiny, and shared decision-making was perceived as well suited to regulate unforeseen circumstances:

But I think again you would build that into your ethics procedure, so that you would say to any of your potential participants, the study is about this, however, these techniques can also reveal other conditions that, health conditions that might have a bearing on your ability to function. And therefore, you know, you’d make it part of the consent process […].

researcher, education

### Psychosis, neurobiological information, and mental health care

I asked researchers and health professionals about the clinical translation of neurobiological findings and the moral challenges that may arise from this translation. Participants expressed polarised views. Interestingly, such views correlated more with participants’ background—whether this was psychiatry, psychology, nursing, or social work—than with their role as researchers or health professionals. The current lack of clinical applications, especially with regard to genomics, was perceived as a moral challenge in itself. However, several participants expressed positive views regarding potential future applications particularly with reference to (1) psychosis prevention, (2) revision of diagnostic categories, and (3) better treatment options and more targeted clinical triage:

I think it’s got to be into stratification. So, it’s got to be into profiling people at first episode of psychosis and really understanding in detail, the whole range of different things that are going on and that’s on the biological, the psychological and the social level. You’ve got to be able to say when a young person comes in front of us, this is the pathway you are likely to take, and this is the treatment that is effective for you. At the moment, we give the same package of treatment to everybody, we have no way of sub-dividing essentially. So, that’s got to be the way forward. We are still so far off that I think we’ve got to use all the tools, all the neuroscientific methods we have available to us to try and do that stratification.

researcher, psychiatry

At the same time, several participants in both groups argued that neurobiology-based diagnostic measures might eventually be harmful. First is the issue of resources. Mental health services were described as structurally under-resourced. Translating new technologies into mental health care might prove difficult because of lack of funding and, at worst, could risk affecting existing resources. Second, researchers highlighted potential risks of over-diagnosis and over-treatment. Several health professionals also noted that misdiagnosis of psychotic illness is part of the history of psychiatry. Hence, it is important to ensure that the translation of neuroimaging and genomics into clinical care does not exacerbate over-diagnosis, but that it is directed towards reducing misdiagnosis. Third, when asked about the potential effects of neurobiology-based diagnostic tools on clinical practice, several health professionals linked the risks of over-diagnosis and over-treatment with the issue of medicalisation:

But then, that takes away an individual's personal choice to access a service and we may be identifying people who don't actually have a difficulty, have unusual experiences, but they're not distressed by them. So why would we bother them? From my point of view, it wouldn’t really be helpful because in most cases people seek help and it's through the assessment of the difficulties that we identify, which services and which interventions may be most helpful.

health professional, psychotherapist

According to several health professionals, potential harms also include (1) the development of more invasive diagnostic measures, which in turn is linked to risks to privacy and confidentiality, and (2) the fact that neurobiological information revealing psychosis-risk status might not be actionable, thus increasing the risks of hopelessness and disengagement in patients and service users:

I think again it's going to depend on the context I think, and on how accurate those predictions might be and then on again what the potential for change would be given you know those risk factors. And, you know if there are likely to be interventions that are useful, otherwise I think the potential for harm probably outweighs the benefit.

health professional, psychiatrist

Participants also argued that the translation of neurobiological findings into clinical care might be welcomed by practitioners depending on their professional background and on the aetiological model of mental illness to which they refer. Professionals with a medical or psychiatric background could respond positively. Conversely, practitioners whose background is in psychology or social work could react with scepticism, and this could generate tensions within clinical teams. However, several participants highlighted the relevance of constructive interplay within multidisciplinary teams. They argued that such interplay would be vital to ensure that the focus of clinical translation remains improving patient care:

So there's a really healthy tension I think with the mental health services between the medically-trained colleagues who have very much an appreciation of the biology and the science involved in the development of mental illness and then the psychologically-trained staff who have very much an appreciation of the psychosocial impact on development of mental health problems. And there's a very healthy tension I think between them that actually creates good care for the patients.

health professional, mental health nurse

Novel diagnostic tools for psychosis could include, for instance, neurobiological markers of psychosis vulnerability and treatment response [5] or machine learning applications to identify psychosis risk and predict psychosis transition or psychotic relapse [11, 34–36]. I asked professionals what might be the ethical implications of using such tools in clinical care. Again, they described potential benefits and potential harms. Interestingly though, participants did *not* frame the harm-benefit discourse around the evaluation of particular diagnostic tools. Rather, they discussed harms and benefits in relation to how the delivery of neurobiological information relating to psychosis is *enacted* and in relation to how clinical decision-making may actively involve patients and service users:

It's all about how that information–, how that conversation is had with the service user, you know. "We want to give you a brain scan because we think it might give us information about your illness" is different to having a conversation with the service user about look, there may be different ways of understanding your experiences, we've taken some blood tests, you know, we might be able to offer you a scan, we'd like to do some talking assessments how do you feel about that? There's different ways of having that conversation with the patient.

health professional, mental health nurse

No, that's the thing, so I think yes I suppose it's how the information that is used isn't it? It might not–, it might be used by people who are providing care as a way of preventing people developing serious mental illnesses, and then the information could be used in an adverse way whereas people are discriminated against.

health professional, mental health nurse

In other words, novel diagnostic tools were perceived as potentially harmful or potentially beneficial in relation to the *degree* of effective communication that is established with the care recipient. This was evident in the discussion around psychosis risk communication. Participants argued that communication of psychosis risk has the potential to increase distress in (asymptomatic) individuals, or even to result in over-treatment:

And it could–, people running away saying ‘I don't want to think about this ever, go away, I don't want to see you’. Or, it could have the opposite effect, it could make people over worried and over anxious and be seeking, you know, consults over the very mildest of symptoms. And maybe the over prescription of medications.

health professional, psychiatrist

Psychosis risk communication should highlight that an increased risk of psychosis does not mean that psychosis transition is inevitable; that psychotic experiences are common in the general population; and that many people are not distressed by them. Overall, several health professionals supported the argument that novel diagnostic tools could be beneficial to patient care *only* if effective clinician–patient communication is established:

I think it's how you talk to the person and help them to understand that all of our brains have to be different, every last human, and this is how your brain actually works, but this is how I can help that brain to give you happiness and a good quality of life.

health professional, counsellor

### Identity, relationships, and the future

I asked both groups how they thought neurobiological explanations of psychosis might relate to Essentialist Thinking (ET) in understanding mental illness and to stigma and labelling. When asked about ET—that is genetic essentialism, or the view according to which specific mental traits emerge inevitably from a genetic ‘essence’, and neuro-essentialism, which is the same view with reference to neural substrates [37, 38]—most researchers said that they had encountered some form of essentialism within their professional network but that ET was *not* common in patients. Participants in both groups agreed that there is a great variation in how patients understand the aetiology and the nature of psychosis. At the same time many health professionals recognised their responsibility in shaping their clients’ views around mental illness:

[…] especially psychiatrists have a great influence over the way people actually think about their illness. So, if they've worked with a psychiatrist that's biologically based in nature then they're more likely to see that their illness is something that's inherent within them. And as an illness, if they've worked with a psychiatrist and mental health practitioners such as nurses that take a much broader view, that look at it as a bio/psycho/social model then they will see that different things cause their illness to come back.

health professional, mental health nurse

Participants expressed a variety of views about what they thought the relationships between the development of neurobiology and essentialism might entail. According to several participants in both groups, focusing on the genomics of psychosis could increase ET in clients and society. At the same time, many researchers argued that *molecular* genomics could in fact reduce ET, by providing evidence that psychotic disorders are complex conditions and that other risk factors play a significant role in psychosis onset. Participants in both groups argued that neurosciences also have the ambivalent potential to increase or reduce ET, depending on *how* information on neurobiology is delivered. Further, both researchers and health professionals recognised that neurobiological models of mental illness have the potential to be either stigmatising or de-stigmatising. For instance, framing the neurobiology of psychosis within a ‘broken brain model’ could reinforce stigma. On the other hand, neurobiology could help to reduce social stigma (1) by removing individual responsibility and blame over the illness and showing that mental illness can impair agency, and (2) by providing a better understanding of the illness and more accurate diagnostic procedures. Interestingly, some professionals refused to describe stigma as linked to aetiological models. They preferred to describe stigma as a social and cultural issue which characterises the diagnostic category of schizophrenia:

I don't think it's related to stigma, I don't think those explanations affect stigma. I think pre-existing knowledge and misinformation affect stigma.[…] Stigma to me is something that's perpetuated by society and not by causation, it's a social difficulty, stigma, not–, nothing to do with biological explanations.

health professional, psychotherapist

Interestingly, several participants in both groups expressed concern over how having access to information on genetic predisposition and brain processes could affect patients and families. Participants argued that neurobiological explanations of psychosis might influence how individuals construct their self-narratives and shape their own identities. This could have positive or negative consequences. On the one hand, most participants agreed that integrating psychosis into one’s own identity might have positive consequences and promote resilience. On the other hand, some researchers highlighted the potential for neurobiological models of psychosis to instil a sense of hopelessness towards recovery:

And if it’s something neuroscientific, then that implies there’s not much you can do yourself that can change that. It’s something wrong with your brain. It’s not something that practicing mindfulness is going to help much. You see what I mean? It’s quite… It doesn’t instil much hope if you’ve a feeling it’s a biological problem.

researcher, psychiatry

Linked to this argument was the idea, expressed by several health professionals, that neurobiological explanations of psychosis might undermine the sense of *agency* and the potential for *recovery* in patients and service users. Most importantly, participants in both groups argued that promoting a neurobiological understanding of psychosis might influence clients’ life choices. Again, this could have positive consequences—if for instance individuals refrain from behaviour that could increase their risk of developing psychosis, such as taking recreational drugs—or negative consequences. Participants expressed concern over the potentially negative, life-limiting influence over clients’ life choices. This concern was evident with regard to reproductive choices:

[It] may impact people's relationships and life choices, so if they feel these are my genes, these is the way I am, it might impact on someone whether to have children or not.

health professional, care coordinator

Impact on family relationships was also recognised as an area of concern. Participants described the risk that families might assume a paternalistic role whereby a (young) person is seen on an inevitable trajectory towards mental illness and therefore her freedom and autonomy are restricted. Interestingly, health professionals highlighted the risk that promoting a neurobiological understanding of psychosis might generate family conflicts by instilling feelings of *blame* and *guilt* towards the illness among family members.

Lastly, I questioned health professionals regarding the possibility of having genetic testing for psychosis and schizophrenia in the future. I reminded participants that such testing does not currently exist. I did not specify what *type* of testing would be available in this thought experiment—whether this would be carrier, prenatal, predictive, or diagnostic testing. Interestingly, participants often expressed negative views when they linked genetic testing to the reproductive domain. Most health professionals expressed concern over the possibility to have carrier or prenatal testing. They feared that such tests would negatively condition individuals’ reproductive choices:

If you identified genetic markers, at what point does that end? So, do you test parents before they start a family to see if they're carriers, to advise them that there's a risk that they might pass on a gene to children potentially? That–, it's huge.

health professional, psychotherapist

[…] and the danger is–, say if it was part of the test you had during scans in pregnancy, the danger is you would use that information to decide whether you're going to have that child or not, and it's kind of quite a skewed picture.

health professional, social worker

However, the group of health professionals expressed ambivalent views regarding predictive or diagnostic testing. On the one hand, many professionals feared that genetic testing might generate hopelessness towards recovery, discrimination of individuals who have genetic predisposition to psychosis, and risk of family conflicts due to feelings of blame and guilt. Conversely, other professionals pictured a narrative of empowerment whereby predictive and diagnostic testing might produce personal benefits. According to this narrative, knowing about genetic predisposition could help individuals to direct their life choices towards psychosis *risk reduction*:

I think it's going to depend on their own health beliefs. Some people may view it as enlightening, they may feel informed, they may feel that yes, they have to make some modifications to their life in order to reduce their risk of developing psychosis.

health professional, mental health nurse

The potential clinical utility of predictive testing was particularly reaffirmed when asymptomatic or help-seeking individuals have a long family history of mental illness. But again, most participants stressed the importance of clinicians’ gate-keeping function in accessing information on genetic predisposition to psychotic illness.

## Discussion

Even though relevant differences persist on the epistemological value attributed to neurobiology across the aetiological divide, this study suggests that the moral challenges of accessing neurobiological information in the context of psychosis reach *far beyond* the traditional dispute between biological and psychosocial approaches to mental illness. Further, this study suggests that whilst they may differ with regard to the recognition of moral obligations pertaining to their professional role, researchers and health professionals from diverse backgrounds recognise similar accounts of the moral challenges and ethical principles governing the acquisition, communication, and use of neurobiological information with individuals who (may) suffer from psychosis. The key message that emerged from the interviews is that information around genomic and brain correlates of psychosis, as well as information around psychosis risk status and illness susceptibility is a *powerful tool* in the process through which research participants and care recipients define their identity and establish personal and clinical goals. A growing body of literature recognises the importance of investigating stakeholders’ perspectives on the expansion of psychiatric genomics and the ethical issues thereof [39, 40], as well as on the translation of neuro-technology in mental health care [41, 42]. This study sits within this debate by describing researches and health professionals’ perceived moral responsibility in managing access to neurobiological information in the context of psychosis which, amongst mental health conditions, has historically been one of the most controversially debated across the aetiological divide [20].

To cite the work of Emily Postan, neurobiological information can be seen as a “tool of narrative self-conception” [19]. In the case of psychosis this tool has profound implications on how individuals see themselves, for instance as being inherently flawed or able to integrate psychotic experiences in their self-image; on how individuals see their actions and choices as restricted by their biological essence or as open to hope, resilience, and recovery; and on how individuals shape their interactions, for instance in establishing conflicting or harmonious relationships with care providers and family members. This argument resonates with psychological theories around *narrative identity* and its relevance for mental health [43]. Further, whilst the development of a coherent personal narrative is essential to mental health, it acquires particular prominence in the context of psychosis [44, 45]. Within this framework, the present study suggests that the *acquisition* of neurobiological information may be morally relevant and yet, not particularly problematic—so long as it respects the rights, dignity and autonomy of research participants and care recipients. Conversely, the way in which neurobiological information operates as a tool of narrative self-conception depends on *how* neurobiological information is communicated and used, in research as in clinical care.

Let me explain this further. As one participant in this study poignantly phrased it, “it’s all about delivery!” [researcher, clinical psychology]. Substantive moral challenges—that is, occurrences that are perceived as morally complex—emerge when information around genetic predisposition and brain processes is delivered to research participants and care recipients. Researchers and health professionals supported the argument that substantive moral challenges do not arise from how neurobiological information is *acquired*, provided that ethical safeguards—such as informed consent of capacitous individuals, thorough ethical review, and specific guidelines to deal with unforeseen situations such as incidental findings—are put in place. Conversely, they suggested that substantive moral challenges arise from how neurobiological information is communicated, how information shapes clinical interventions and social interactions, and how information affects self-narratives and decision making. I shall briefly explain how this argument relates to the three thematic areas presented in the results.

First, research ethics. Neither researchers nor health professionals expressed relevant concerns around traditional research ethics issues. These were seen as important but largely unproblematic. Researchers and health professionals distanced themselves from paternalistic, population-based accounts of vulnerability [46, 47]. They framed access to neurobiological research around autonomy and non-discrimination. This resonates with a body of literature which highlights how individuals who suffer from mental illness often insist upon their equal right to participation [48]. Further, most researchers in this study recognised that vulnerability can stem from certain factors—such as age, capacity to consent, or clinical status—but did not believe individuals who suffer from psychosis require additional protection *only* because of their diagnosis. This echoes Bracken-Roche and colleagues’ critique of class membership accounts of vulnerability as ill-suited to represent the reality of psychiatric research participants [49]. Again, researchers recognised that they have a moral obligation to return aggregate or even individual results where participants wish to know them, and that they have a duty to communicate clinically actionable incidental findings [17, 50] while avoiding therapeutic misconception [51]. To summarise, traditional research ethics issues were perceived as easily solvable via REC / IRB review, professional guidelines, and shared decision-making. At the same time, the ‘delivery’ of neurobiological information to research participant was problematized. In other words, *whether* to enrol individuals with psychosis in neurobiological research or whether to disclose results were perceived as morally unproblematic. Conversely, *how* to communicate neurobiological findings was perceived as morally problematic.

Second, participants pictured a similar narrative about the translation of neuroscience and genomics into mental health care. Health professionals framed the discourse around the harms and benefits of neurobiology-based diagnostic tools in relation to the *degree* of effective communication established with care recipients. This was evident with reference to psychosis-risk communication and psychosis prediction [52, 53]. Health professionals expressed concerned about having a positive or negative impact on their clients’ (developing) identity. Whether such an impact might be positive or negative depends on how neurobiological information is ‘delivered’—that is, communicated or used—in the clinical encounter. This study suggests that delivering neurobiological information on psychosis in clinical care can *potentially* be beneficial or harmful to the development of clients’ identity. The *actual* effect on clients’ identity depends on the modalities of such delivery. For instance, Kong et al. have highlighted the risk that genomic medicine might promote fatalism towards mental illness, which in turn could undermine patients’ agency and autonomy [54]. This argument closely relates to the idea of hopelessness described by health professionals in this study. Whether delivering information on genomics and brain processes might promote fatalism and instil a sense of hopelessness or, conversely, be positively incorporated in a client’s personal narrative depends on *how* such delivery is enacted. Again, this resonates with recent literature on interview-based risk assessment and psychosis prediction, which highlights the importance of developing appropriate communication strategies and of promoting a narrative of empowerment against a sense of hopelessness in care recipients [52, 55].

Third, this study suggests that the relations between neurobiological information and identity are much broader than the ethico-legal implications captured by the discourse around benefits and harms in research and care. For instance, it is not clear whether biogenetic explanations of psychosis might increase or reduce stigma and self-stigmatisation, as the relations between biogenetic explanations, essentialist thinking, and stigma are extremely complex [42, 56–58]. Participants in this study corroborated this view. However, they emphasised researchers and clinicians’ responsibility in shaping views around psychosis and in contrasting stigma [59]. Further, health professionals suggested that there are situations in which having predictive genetic testing could have clinical or personal utility by directing life choices towards risk reduction, particularly when individuals who could undergo predictive testing have a family history of mental illness. Rather than establishing precise criteria to evaluate the utility of psychiatric genetic testing, such framing highlights practitioners’ responsibility in mitigating the potentially negative ramifications of genetic testing for patients and families [60] while promoting (non) medical benefits which could result from predictive testing [61]. Overall, practitioners’ gate-keeping function seemed to be constrained by their perceived moral obligation to ensure that neurobiological information on psychosis can positively affect clients’ self-narratives and personal identities.

### Limitations

This study is situated in the domain of consultative approaches to empirical bioethics as it constitutes what De Vries has called descriptive ethics or sociology *in* bioethics [24, 62]. No strong normative claims are grounded in the study results. Yet, as medical technologies are translated into psychiatry, providing a snapshot of the moral life of the actors who must deal with this translation can help bioethicists to frame the normative discourse around ethical principles and moral obligations [23, 63]. This article has three limitations. First, the perspectives of the most relevant actors in mental health care—that is, patients and service users—were not investigated in the ELSI-NAPS study. The reason for this is to be found in the study rationale: given that the ethico-legal debate is particularly focused on the identification of principles and obligations that pertain to professionals in research and care, ELSI-NAPS aimed at investigating these actors’ perspectives. This fact does *not* entail that patients and service users’ perspectives are not important or not worthy of qualitative investigation. Rather, the findings presented in this article highlight the need for further investigation to explore the perspectives of patients and service users on the moral challenges of accessing neurobiological information in the context of psychosis. Second, this article provides a qualitative overview, but given the nature of purposive sampling the results cannot be generalised to the population of researchers and mental health professionals. The decision to adopt purposive sampling was again based on the study rationale, which was to investigate a *variety* of professional viewpoints across the aetiological divide at the expenses of generalisability. The third limitation relates to bias in the perception of moral challenges. Professional backgrounds as reported in the demographics and the cultural *milieu* surrounding participants—recruitment took place in community services and in universities in England—likely influenced perception of moral challenges. I believe that situating participants’ perception of moral challenges within a specific social context does *not* amount to evidencing bias in participants’ views. Rather, it highlights that moral principles and obligations are often embedded within a person’s lived experience, and this experience might be of epistemic value for empirical bioethics [64]. I believe that these limitations do not invalidate the study results and that declaring them can better situate the analysis.

## Conclusions

Several scholars argue that translating neuroimaging and genomics into psychiatry is imperative, given the burden of mental illness on population health [9, 65]. Conversely, psychosocial researchers often criticise the expansion of neurobiological approaches to psychosis on ethical and even political grounds [21]. As an exercise of aetiological neutrality, this study suggests that the ethical implications of biomedical innovation in psychiatry may go beyond this, even though extremely important, normative issue. More precisely, this study suggests that the ethical ramifications of accessing neurobiological information in the context of psychosis reach far beyond the sound conduct of clinical research and the ethical translation of research findings in mental health care. Provided that these two activities are carried out by respecting the rights, dignity, and autonomy of those who (may) suffer from psychosis, the actors who perform such activities—that is, researchers and mental health professionals—are likely to recognise that moral obligations towards their clients extend to the *identity impacts* that accessing neurobiological information can have in the context of psychosis. As the very actors who operate this tool of narrative self-conception, researchers and mental health professionals will need ethical guidance on how to operate such a powerful instrument.

Researchers and health professionals share a moral responsibility in shaping views around mental illness and recovery. Recognising this responsibility might be a first step towards ensuring that they can face the moral challenges of their professional role. First, it will be important to implement non class-membership accounts of vulnerability [49, 66] which must balance the need to protect individuals from the risks of research participation with the demand for fair access to research, and the respect of the autonomous choices of those (capacitous) individuals who wish to take part in research, access results, and know incidental findings. Second, ensuring that the translation of neuroimaging and genomics into mental health care is beneficial to patients will require health professionals to reflect not only on whether to disclose neurobiological information, but also on *how* this is communicated and *how* it might shape clinical decision-making and social relationships. Third, if neurobiological information is a tool of narrative self-conception, it will be important to develop appropriate guidance on how to use such a tool so that accessing neurobiological information may be beneficial and not detrimental to the development of individual self-narratives. A tool is often morally neutral but can acquire a moral connotation from the ways in which it is used. The way in which individuals who (may) suffer from psychosis construct their self-narratives, define their own identities, and cultivate social relationships is vital to their recovery journey [44, 67]. Hence, reflecting on how accessing information on genetic predisposition and brain processes can affect people’s narratives will be vital in order to ensure that neuroscience and genomics can truly benefit those who experience psychosis. Further research, both normative and empirical, is needed to establish not only whether but also how neurobiological information ought to be delivered in the context of psychosis.

## Supplementary information

**Additional file 1**. Interview guide researcher (Group A).

**Additional file 2**. Interview guide mental health professional (Group B).

**Additional file 3**. Coding manual researchers (Group A).

**Additional file 4**. Coding manual mental health professionals (Group B).

## Data Availability

The datasets generated and analysed during this study are not publicly available because they contain information that could compromise the privacy of research participants. The data may be made available by the corresponding author [PC] upon a reasonable request from a bona fide researcher in order to validate the reliability of data analysis.

## Abbreviations

ET: Essentialist thinking
GCP: Good clinical practice
IRB: Institutional Review Board
NHS: National Health Service
REC: Research Ethics Committee
