# Supplementary material for: “It’s all about delivery”: researchers and health professionals’ views on the moral challenges of accessing neurobiological information in the context of psychosis
Source: BMC Med Ethics. 2021 Feb 8;22:11. doi: 10.1186/s12910-020-00551-w (PMC7869514; doi:10.1186/s12910-020-00551-w)
Supplement: Supplementary file 1 — Additional file 1. Interview guide researcher (Group A). [file 12910_2020_551_MOESM1_ESM.docx]

**Interview Guide: Researcher (Group A)**

| **Themes** | **Questions** |
| --- | --- |
| **Warm up** | - For how long have you been working as a researcher in psychology / psychiatry / mental health? - In few words, how would you describe your main research field? - Over the course of you career, have you been performing clinical research on psychotic disorders? What kind of research have you performed?   In recent years, the development of medical technologies such as neuroimaging and whole genome sequencing has resulted in an increased interest in the neurobiology of psychosis and schizophrenia. As we know, psychotic disorders are best understood in a *bio-psycho-social* model, as they involve at the same time biological, psychological and social factors.   - Overall, what are your thoughts on the increased research interest in the neurobiology of psychotic disorders? - What do you feel could be the arguments for justifying conducting this kind of research? |
| **Ethical Issues:**  **Clinical research** | Some ethical and legal issues that may occur while performing *neuroscience* and *genomic* research with clinical populations have been identified. I would like to know your opinion on those.   - What are your thoughts on returning results from neuroimaging and genomic studies to research participants with a psychotic disorder? - Would you consider it *beneficial* to return results to a clinical population with a psychotic disorder? Why / Why not? - How would you behave if significant Incidental Findings (IFs) on a participant resulted from your research? Has this happened to you before? How have you reacted? - Neuroimaging studies can incidentally highlight underlying brain conditions. Would you communicate these to participants? In what circumstances? How? - Would you involve family members / carers / significant others? - Genomic studies can generate IFs that can be relevant to family members, especially regarding the risk of developing specific conditions. What are your thoughts on this? - One of the strongest ethical concerns about research into the neurobiology of mental illness is the lack of immediate Clinical Utility (CU). What are your thoughts on this? - Do you think that neuroimaging or genomics could support diagnosis / prognosis / treatment of psychotic disorders? Why / Why not? How long will it take before we see CU in your estimation? - Given the lack of immediate CU, do you feel that research into the neurobiology of mental illness is ethically justified? Why / Why not? - How would you communicate lack of immediate CU to participants / family members? - Genomics research aims to uncover the biological basis of vulnerability to psychosis, as well as familial risk. What are your thought on this? What about clinical utility? - In your opinion, what are the ethical challenges of involving patients / service users with psychosis or schizophrenia in genomics research? You can mention as many as you want. - What about young patients / service users or minors? - Do you think that risk assessment based on genetic measures may be *beneficial* to individuals at high-risk or ultra-high-risk of developing a psychotic disorder? Why / why not? - What are your thoughts on recent findings in GWAS studies on psychotic disorders and schizophrenia? - What do you think would be the reasons to perform WGS / WES with patients / service users with a psychotic disorder? - Do you see any ethical concern *not to* perform that? - What do you think may be the ethical implications? - What do you think would be the implications for patients / family members? |
| **Ethical Issues:**  **Common** | Genetic essentialism is defined as a tendency, in the general population, to identify biogenetic explanations of illness as the *only* possible explanations of a particular disorder, and genetic susceptibility / risk as genetic *inevitability* to develop the disorder.   - Do you think that genomic studies on mental illness may / may not contribute to enforce this tendency? Why / why not? - Have you encountered this facet in your professional experience? - Would you say that the clinical populations you work with are / are not prone to genetic essentialism? - What about young / minor populations? - How could researchers contribute to contrast genetic essentialism?   Likewise, neuro-essentialism is defined as the same tendency, with regard to neuroscientific studies on the neurological correlates of mental illness.   - Do you think that neuroscientific studies on mental illness may / may not contribute to enforce this tendency? Why / why not? - Have you encountered this facet in your professional experience? - Would you say that the clinical populations you work with are / are not prone to neuro-essentialism? - What about young / minor populations? - How could researchers contribute to contrast neuro-essentialism? - How do you think that neuroscientific and genomic explanations of mental illness might affect individuals’ vulnerability? - What about young clinical populations? - What about young individuals / minors in the prodromal phase of psychosis? What about their families and carers? - In the case of young individuals / minors, how do you think that neurobiological measures would affect identity formation? - What about early internalisation of genetic and neuro-essentialism? - What about family interactions? - Do you think that neurobiological explanations of mental illness may increase / decrease stigma and labelling at a social level? - At the individual level? - Do you see labelling that may derive from neurobiological diagnostic measures as beneficial / non-beneficial to patients / service users? Why? - Do you think that neurobiological diagnostic measures would increase / decrease self-stigmatisation? Why / why not? - Could you think of any ethical or legal issue that might derive from the introduction of neurobiological diagnostic / prognostic measures to informed consent procedures?   You can refer to clinical research or clinical practice.   - Would you see any major ethical or legal issue arising from the use of neuroimaging or genomics measures in forensic psychiatry? |
| **Potential Clinical Translation & Ethical Issues Involved** | Current translational efforts include: 1) neurobiological markers of vulnerability to psychosis / transition / disease progression; 2) neuro-functional markers in the psychosis prodrome; 3) drug discovery and development; 4) integration of different modalities with machine learning methods for individual prediction of psychosis transition.   - What are your thoughts on the potential clinical utility of the above translational efforts? - Can you think of any ethical concerns that could arise from those? - How do you think measures such as the ones described above may affect patients / service users’ sense of identity? - What about agency or personal autonomy? - What about the risks of stigmatization and labelling? |
| **Impact on Mental Health Care** | - Given the current strong focus on early intervention for psychosis and schizophrenia in mental health services, do you think that the introduction of such measures could affect patients’ clinical outcomes? Why / why not? - What about patients / service users’ engagement with EIS or other community clinical teams? - What about patients being hosted in inpatient units? - What about young / minor service users, and their families? - What about mental health care providers, such as psychologists, psychiatrists and social workers? How do you think they would welcome the potential introduction of such measures? Why? |
