# Supplementary material for: “It’s all about delivery”: researchers and health professionals’ views on the moral challenges of accessing neurobiological information in the context of psychosis
Source: BMC Med Ethics. 2021 Feb 8;22:11. doi: 10.1186/s12910-020-00551-w (PMC7869514; doi:10.1186/s12910-020-00551-w)
Supplement: Supplementary file 2 — Additional file 2. Interview guide mental health professional (Group B). [file 12910_2020_551_MOESM2_ESM.docx]

**Interview Guide: Mental Health Professional (Group B)**

| **Themes** | **Questions** |
| --- | --- |
| **Warm up** | - For how long have you been working as a health care professional in mental health? - In few words, how would you describe your background and your current occupation? - Over the course of you career, have you been working alongside patients / service users with a psychotic disorder or schizophrenia? Can you give me some examples of your involvement with this population?   In recent years, the development of medical technologies such as neuroimaging and whole genome sequencing has resulted in an increased interest in the neurobiology of psychosis and schizophrenia. As we know, psychotic disorders are best understood in a *bio-psycho-social* model, as they involve at the same time biological, psychological and social factors.   - Overall, what are your thoughts on the increased research interest in the neurobiology of psychotic disorders? - What do you feel could be the arguments for justifying conducting this kind of research? |
| **Ethical Issues:**  **Common** | Some ethical issues that may be identified while conducting neuroscience and genomic research with clinical populations can also be encountered in clinical practice. I would like to know your opinion on those.  Genetic essentialism is defined as a tendency, in the general population, to identify biogenetic explanations of illness as the *only* possible explanations of a particular disorder, and genetic susceptibility / risk as genetic *inevitability* to develop the disorder.   - Do you think that genomic studies on mental illness may / may not contribute to enforce this tendency? Why / why not? - Have you encountered this facet in your professional experience? - Would you say that the clinical populations you work with are / are not prone to genetic essentialism? - What about adolescent / child populations? - How could clinicians contribute to resist or combat genetic essentialism?   Likewise, neuro-essentialism is defined as the same tendency, with regard to neuroscientific studies on the neurological correlates of mental illness.   - Do you think that neuroscientific studies on mental illness may / may not contribute to enforce this tendency? Why / why not? - Have you encountered this facet in your professional experience? - Would you say that the clinical populations you work with are / are not prone to neuro-essentialism? - What about adolescent / child populations? - How could clinicians contribute to resist or combat neuro-essentialism? - How do you think that neuroscientific and genomic explanations of mental illness might affect individuals’ vulnerability? - What about young clinical populations? - What about young individuals / minors in the prodromal phase of psychosis? What about their families and carers? - In the case of young individuals / minors, how do you think that neurobiological measures would affect identity formation? - What about early internalisation of genetic and neuro-essentialism? - What about family interactions? - Do you think that neurobiological explanations of mental illness may increase / decrease stigma and labelling at a social level? - At the individual level? - Do you see labelling that may derive from neurobiological diagnostic measures as beneficial / non-beneficial to patients / service users? Why? - Do you think that neurobiological diagnostic measures would increase / decrease self-stigmatisation? Why / why not? - Could you think of any ethical or legal issue that might derive from the introduction of neurobiological diagnostic / prognostic measures to informed consent procedures?   You can refer to clinical research or clinical practice.   - Would you see any major ethical or legal issue arising from the use of neuroimaging or genomics measures in forensic psychiatry? |
| **Potential Clinical Translation & Ethical Issues Involved** | Current translational efforts of research findings include the following:  1) neurobiological markers of vulnerability to psychosis / transition / disease progression; 2) neuro-functional markers in the psychosis prodrome; 3) drug discovery and development; 4) integration of different modalities with machine learning methods for individual prediction of psychosis transition.   - What are your thoughts on the potential clinical utility of the above translational efforts? - Can you think of any ethical concerns that could arise from those? - How do you think measures such as the ones described above may affect patients / service users’ sense of identity? - What about agency or personal autonomy? - What about the risks of stigmatization and labelling? |
| **Ethical Issues: Clinical Practice** | - In your opinion, is there any risk of increasing patients / service users’ self-stigmatising attitudes by implementing novel neurobiological approaches to psychotic disorders? Why / why not? - How do you think that the introduction of measures such as the ones described before would affect your relationship with patients / service users? - In your opinion, would that improve / obstacle engagement? Why? - Would that affect in any way the relation of trust that you establish with patients / service users? Why / why not? - If accurate neurobiological measures for risk prediction (for developing psychosis or schizophrenia) were available, would you be willing to use them / propose them to patients / service users? Why / why not? - How would you communicate the risk of developing a psychotic disorder based on neurobiological measures to patients / service users? What about the family / relevant others? - Would you involve families in communicating the risk of developing a psychotic disorder or schizophrenia? Why / why not? - What about over-diagnosis? In your opinion, could there be a risk of over-diagnosis of psychotic disorders, should neurobiological measures of risk prediction be implemented? Why / why not? - To what extent would you, as a mental health professional, rely on those measures? - In the case of scarcity of resources, on what basis would you allocate them? Following which criteria? - In your opinion, what would be the impact on patients / service users’ families and carers? - What about the young / prodromal population? What about minors? - What about the families / relevant others of chronic patients with a psychotic disorder or schizophrenia?   Genetic Testing (GT) is not currently available for common complex mental disorders such as schizophrenia. Given that psychotic disorders involve at the same time biological, psychological and social factors, some people argue that it will *never* be available, or that it *should not*  available.   - What are your thoughts on this matter? Remember, you can be as honest as you want. - If GT were available for psychotic disorders, would you be willing to use it / propose it to patients / service users? Why / why not? - Would you consider offering genetic counselling? Why / why not? - What impact do you think GT for psychotic disorders could have on patients / services users? - What about the young population? What about minors? - What impact do you think GT for psychotic disorders could have on individuals already identified as at-risk, based on clinical presentation of prodromal symptoms, or on family history? - What impact do you think GT for psychotic disorders could have on their families / relatives? - Should GT be available for psychotic disorders, would you foresee any risks of genetic discrimination based on the results of such tests? - Why / why not? - How would you communicate the results of such tests to patients / services users? And to their families / relevant others? |
| **Impact on Mental Health Care** | - Given the current strong focus on early intervention for psychosis and schizophrenia in mental health services, do you think that the introduction of neurobiological measures could affect patients’ clinical outcomes? Why / why not? - What about patients / service users’ engagement with EIS or other community clinical teams? - What about patients being host in inpatient units? - What about young / minor service users, and their families? - What about mental health care providers, such as psychologists, psychiatrists and social workers? How do you think they would welcome the potential introduction of such measures? Why? |
