# Supplementary material for: “It’s all about delivery”: researchers and health professionals’ views on the moral challenges of accessing neurobiological information in the context of psychosis
Source: BMC Med Ethics. 2021 Feb 8;22:11. doi: 10.1186/s12910-020-00551-w (PMC7869514; doi:10.1186/s12910-020-00551-w)
Supplement: Supplementary file 3 — Additional file 3. Coding manual researchers (Group A). [file 12910_2020_551_MOESM3_ESM.docx]

**Coding Manual, Researchers (Group A)**

**Bold = code / theme**; *Italic = code description*

**ARGUMENTS FOR / AGAINST RESEARCH:**

| Pro: | *Arguments in favor of conducting clinical research on the neurobiological (neuroscience and genomics) correlates of psychosis and schizophrenia.* | | | |
| --- | --- | --- | --- | --- |
|  | **Legitimate area of inquiry:** | *Neuroscience and genomics of psychotic illness are legitimate areas of scientific inquiry, as much as psychosocial research / interventions.* | | |
|  |  | **Informed consent and autonomy argument:** | | *No reason to believe that an individual with psychosis, but who has capacity, should be given special consideration.* |
|  | **Knowledge good in itself** | *Scientific knowledge has value in itself and does not need justification.* | | |
|  | **Duty towards society:** | *Scientists have a moral duty towards society to investigate the neurobiological basis of mental illness.* | | |
|  | **Better diagnosis / Novel treatment:** | *Neuroscience and genomics have the potential to produce novel treatments / medications for psychotic disorders.* | | |
| Against: | *Arguments against conducting clinical research on the neurobiological (neuroscience and genomics) correlates of psychosis and schizophrenia.* | | | |
|  | **Resources:** | *Resources for mental health research / care are scarce.* | | |
|  |  | **High cost:** | *Neurobiological research has high costs.* | |
|  |  | **Drains funding:** | *Neurobiological research drains funding from other types of research.* | |
|  | **No novelty, just new technologies:** | *Neurobiological research is not new. It has been ongoing for decades, and has produced little clinically useful knowledge. What is new is the technology.* | | |
|  | **Psychosocial research has greater therapeutic impact:** | *Research on psychosocial factors / interventions has proven to have greater therapeutic impact.* | | |
|  | **Potential for harmful developments:** | *Neurobiological research has the potential to promote harmful future developments in psychiatry. There are potentially harmful driving forces in psychiatry.* | | |

**RETURNING RESULTS:**

| Context-dependent: | *Returning results to research participant is dependent upon the context of the research (methodology, results) and populations / individuals.* | |
| --- | --- | --- |
|  | **Consider risks / situation:** | *Researchers must consider the risks / situation for research participants.* |
|  | **Tailor communication with participants:** | *Communication of research results must be tailored to the situation / capacity / understanding of participants.* |
| Participants’ capacity: | *If participants are deemed to have capacity to take part in research, they should be offered the possibility to know the results. Anti-discriminatory argument.* | |
| Genomics: more sensitive area: | *Genomic research deals with more sensitive information and research procedures. Careful consideration should be given on whether / how to return results.* | |
|  | **Managing genetic information:** | *Handling / managing genomic information is more complex than neuroscientific information.* |
|  | **Risk of deterministic thinking:** | *There is a risk to promote deterministic think (e.g. genetic predisposition as ‘fate’, ‘destiny’).* |
|  | **Discrimination / Insurances:** | *Genetic information may have impact on health insurance policies (e.g. US health care model).* |

**INCIDENTAL FINDINGS:**

| Participant’s capacity: | *Disclosure of incidental findings to a participant should be considered if the participant has capacity.* | | |
| --- | --- | --- | --- |
| Duty to report / right (not) to know: | *Researchers have a moral duty to report (clinically) relevant incidental findings. Participants retain a right (not) to know incidental findings.* | | |
| Shared decision making: | *Disclosure of incidental findings must be considered through a shared decision-making process involving several actors.* | | |
|  | **Other professionals:** | *Involve other professionals (GP, psychiatrist, colleagues) in the decision-making process.* | |
|  | **Family:** | *Consider involving the participant’s family in the decision-making process.* | |
|  |  | **Always consult family for minors:** | *The family should always be consulted in case of minors.* |
| Refer to protocol / guidelines: | *Most of the ethical-legal dilemmas related to disclosure of incidental findings can / must be resolved by referring to the research protocol and / or established guidelines.* | | |
| Avoid therapeutic misconception: | *In considering / disclosing incidental findings, researchers must be aware of the risk that participants might see the researcher as having a clinical, rather than a research role.* | | |

**LACK OF CLINICAL UTILITY (CU):**

| Ways to respond to / communicate lack of CU: | *Ways to conceptualize and justify the lack of immediate clinical utility of neurobiological research. Ways to communicate this lack of CU to research participants.* | |
| --- | --- | --- |
|  | **Intrinsic value of science:** | *Science and knowledge production have intrinsic value.* |
|  | **Honesty & transparency:** | *Researchers must be honest and transparent regarding lack of CU.* |
|  | **Good communication with participants:** | *Researchers must establish good communication with participants, so that they can communicate the lack of CU.* |
| Genomics has less potential CU: | *Genomic science has had, and still has less potential clinical utility if compared with other areas of clinical research.* | |
|  | **Molecular genomics has greater potential:** | *Novel molecular genomics, including WGS has greater potential CU than traditional genomics.* |
| Hope for benefits in the future: | *Lack of immediate CU is balanced by the hope for potential future clinical benefits, which may derive from current research.* | |
| Psycho-social approaches have greater CU: | *Psychosocial research has had and still has more clinical utility.* | |

**ESSENTIALIS THINKING (ET):**

| Clients vs. professionals: | *Clients (e.g. service users, patients, research participants) and professionals (clinicians and researchers) hold different views regarding mental illness.* | | |
| --- | --- | --- | --- |
|  | **ET more common in professionals:** | *ET is more common in professionals (with a neurobiological background) rather than in clients.* | |
|  | **ET less common in clients:** | *ET is generally not common in clients (psychosocial explanations of mental illness are more common).* | |
|  |  | **Great variation in clients:** | *Clients show a great variety of visions regarding the origin / causes / nature of mental illness.* |
| Genomics: | *Genomics and ET.* | | |
|  | **Increase ET:** | *Genomic science may increase ET. Genes as ‘destiny’, ‘fate’. Historical implications of genomic science for mental illness.* | |
|  | **Reduce ET:** | *Development of genomic science may reduce ET.* | |
|  |  | **Complex conditions:** | *Molecular genomics reveals that psychotic illness is complex, non-mendelian.* |
|  |  | **Other risk factors:** | *Molecular genomics reveals that genetic predisposition interacts with other risk factors (e.g. gene-environment interaction).* |
| Neuroscience: | *Neuroscience and ET.* | | |
|  | **Increase ET:** | *Neuroscience may increase ET (e.g. psychosis and schizophrenia understood as pure brain disorders).* | |
|  |  | **Broken brain model:** | *Neuroscientific findings may boost the idea of mental illness due to a ‘broken brain’. This may increase ET.* |
|  |  | **Over-represented by media:** | *Neuroscientific findings are over-represented in the media, if compared with psychosocial approaches. This may increase ET.* |
|  |  | **Positive effects of ET:** | *ET may have positive effects on the way individuals frame their responsibility towards their acts and illness.* |
|  | **Reduce ET:** | *Better understanding of neurobiological ‘correlates’ of mental illness could reduce ET.* | |
| Ways to contrast ET: | *Ways in which researchers could contribute to contrast ET.* | | |
|  | **Educating the public:** | *Researchers should have a role in educating the public about the real implications of the neuroscience of mental illness.* | |
|  | **Better dissemination / communication:** | *Researchers should promote clearer dissemination of research findings and better communication with the media.* | |
|  |  | **Biological ‘factors’, not ‘causes’:** | *Researchers should educate the public about the fact that biological ‘factors’ are not direct / only ‘causes’ of mental illness.* |

**IMPACT:**

| On self: | *Neurobiological explanations of psychotic illness have an impact on self / identity.* | | |
| --- | --- | --- | --- |
|  | **Illness rejection & externalization:** | *Mental illness viewed as something ‘external’ to self (e.g. brain disorder / chemical imbalance) and thus: 1) rejected, or 2) externalized. Positive and negative consequences.* | |
|  | **Illness integration:** | *Mental illness viewed as something part of self (e.g. my brain, my genes, my biology), and thus integrated within the identity. Mostly positive consequences.* | |
|  |  | **Promote resilience:** | *Illness integration promotes resilience (e.g. learning how to accept symptoms, how to deal with symptoms, instead of aiming for full remission).* |
|  | **Risk of hopelessness:** | *Neurobiological explanations of mental illness may promote hopelessness in clients (e.g. psychosis as permanent, chronic condition).* | |
| On families: | *Neurobiological explanations of psychotic illness impact on families of (young) people.* | | |
|  | **Risk of paternalistic role:** | *Within a biomedical model of psychotic illness, families may tend to ‘control’ a young person with psychosis, and assume a paternalistic role.* | |
| On life choices: | *A neurobiological understanding of psychotic illness may impact on individual’s life choices, in a positive way (avoid exposing oneself to risk factors, e.g. cannabis), or negative way (avoid pursuing certain life choices, e.g. education).* | | |
|  | **Reproductive choices:** | *A neurobiological understanding of psychotic illness may impact on an individual’s reproductive choices (e.g. wish not to pass this condition to one’s offspring).* | |

**STIGMA & LABELLING:**

| Psychosocial models: | *Impact of psychosocial models of psychotic illness on social stigma and labelling.* | | |
| --- | --- | --- | --- |
|  | **De-stigmatising:** | *Psychosocial models decrease social stigma.* | |
|  |  | **Blame social factors:** | *Psychosocial models of psychotic illness reduce self-blame, as they focus on social risk factors.* |
|  |  | **Promote hope and empowerment:** | *Psychosocial models of psychotic illness promote hope towards recovery, and promote individual empowerment.* |
| Neurobiology: | *Impact of neurobiological models of psychotic illness on social stigma and labelling.* | | |
|  | **Stigmatising:** | *Neurobiological models increase social stigma.* | |
|  |  | **Broken brain model:** | *The ‘broken brain’ model creates a category of ‘different / diverse’ individuals.* |
|  |  | **Reduced potential for recovery:** | *A neurobiological understanding of psychotic illness reduces the potential for recovery (permanent, chronic condition).* |
|  | **De-stigmatising:** | *Neurobiological models decrease social stigma.* | |
|  |  | **Illness removes responsibility:** | *Psychotic individuals are not ‘responsible’ for their illness / condition.* |
|  |  | **Illness impairs agency:** | *Psychotic individuals are not always / fully in control of their actions, therefore they are not to blame / stigmatise.* |
|  |  | **More accurate diagnosis:** | *More accurate diagnostic procedures based on neurobiology reduce stigma.* |
|  |  | **Better understanding of illness:** | *Better understanding of the condition (as a medical disorder) removes stigma (e.g. AIDS, cancer, dementia).* |

**CLINICAL TRANSLATION:**

| Potential benefits: | *Translating findings from neuroscience and genomics into clinical care may produce potential benefits.* | | | |
| --- | --- | --- | --- | --- |
|  | **Prevention:** | *Neuroscience and molecular genomics may positively impact on prevention of psychosis and schizophrenia (e.g. by identifying at-risk populations).* | | |
|  | **Change diagnostic system:** | *Neuroscience and molecular genomics may radically change our (already broken) diagnostic system.* | | |
|  | **Better treatment / medications:** | *Neuroscience and molecular genomics will produce novel and better treatments / medications for psychosis and schizophrenia.* | | |
| No impact: | *Neuroscience and genomics will have no substantial impact on how clinical care is delivered.* | | | |
| Potential harms: | *Translating findings from neuroscience and genomics into clinical care may result in risk of harm for (young) clinical populations.* | | | |
|  | **Risk of over-diagnosis / overtreatment:** | *Using neuroscience and genomics in clinical care increases the risk of over-diagnosing and thus over-treating young (healthy) individuals.* | | |
|  | **Scarce resources:** | *Resources for mental health care are already scarce.* | | |
|  |  | **Impact on existing resources:** | | *Novel biomedical technologies may impact on current resource allocation.* |
|  |  | **No money for new technologies:** | | *Mental health care shows a structural scarcity of resources to support (fair) access to new technologies.* |
| Impact on services: | *Impact on current mental health services.* | | | |
|  | **Possible professional conflict:** | | *Different professionals will probably react in very different ways, thus possibly generating moral / ethical conflicts.* | |
|  | **Clinicians’ response:** | | *Reaction of mental health professionals to the introduction of neuroscience / genomic based diagnosis / treatment.* | |
|  |  | | **Based on their background:** | *Clinician’s reaction may be affected by their professional background.* |
|  |  |  | **Scepticism:** | *Most mental healthcare providers may be sceptical of the clinical utility of translational efforts.* |
