# Supplementary material for: “It’s all about delivery”: researchers and health professionals’ views on the moral challenges of accessing neurobiological information in the context of psychosis
Source: BMC Med Ethics. 2021 Feb 8;22:11. doi: 10.1186/s12910-020-00551-w (PMC7869514; doi:10.1186/s12910-020-00551-w)
Supplement: Supplementary file 4 — Additional file 4. Coding manual mental health professionals (Group B). [file 12910_2020_551_MOESM4_ESM.docx]

**Coding Manual, Mental Health Professionals (Group B)**

**Bold = code / theme**; *Italic = code description*

**ARGUMENTS FOR / AGAINST RESEARCH:**

| Pro: | *Arguments in favour of conducting clinical research on the neurobiological (neuroscience and genomics) correlates of psychosis and schizophrenia.* | | |
| --- | --- | --- | --- |
|  | **Legitimate area of inquiry:** | *Neuroscience and genomics of psychotic illness are legitimate areas of scientific inquiry, as much as psychosocial research / interventions.* | |
|  |  | **Informed consent and autonomy argument:** | *No reason to believe that an individual with psychosis, who has capacity, should be given special consideration. NO DISCRIMINATION argument.* |
|  | **Knowledge good in itself:** | *Scientific knowledge has value in itself and does not need justification.* | |
|  | **Better understanding of illness:** | *Neurobiological research is essential to reach a better understanding of psychotic illness.* | |
|  | **Better diagnosis / Novel treatments:** | *Neuroscience and genomics have the potential to produce novel treatments / medications for psychotic disorders.* | |
| Against: | *Arguments against conducting clinical research on the neurobiological (neuroscience and genomics) correlates of psychosis and schizophrenia.* | | |
|  | **Drains funding:** | *Neurobiological research drains funding from other types of research.* | |
|  | **Potential for harmful developments:** | *Neurobiological research has the potential to promote harmful future developments in psychiatry. There are potentially harmful driving forces in psychiatry.* | |
|  |  | **Negative impact on clients:** | *Neuroscience and genomics could have a negative impact on clients’ self.* |
|  | **Lack of CU:** | *Neurobiological research has traditionally produced few useful treatments / interventions.* | |
|  |  | **Psycho-social research has greater CU:** | *Research on psychosocial factors / interventions has proven to have greater therapeutic impact.* |

**ESSENTIALIST THINKING (ET):**

| Great variation in clients: | *Clients show a great variety of visions regarding the origin / causes / nature of mental illness.* | | |
| --- | --- | --- | --- |
|  | **Clinicians’ responsibility in shaping views:** | *Clinicians hold great responsibility in shaping patients and service users’ views on the nature of their mental illness.* | |
| Genomics: | *Genomics and ET.* | | |
|  | **Increase ET:** | *Genomic science may increase ET. Genes as ‘destiny’, ‘fate’. Historical implications of genomic science for mental illness.* | |
|  |  | **Fatalistic approach / Inevitability:** | *Genomic science may instil a fatalistic approach toward illness = illness as inevitable.* |
|  |  | **Risk of hopelessness:** | *Genomic science may instil loss of hope towards recovery.* |
| Neuroscience: | *Neuroscience and ET.* | | |
|  | **Increase ET:** | *Neuroscience may increase ET (e.g. psychosis and schizophrenia understood as pure brain disorders).* | |
|  |  | **Deterministic / medical approach:** | *Neuroscience could boost a deterministic medical approach to psychosis, which does not consider social and psychological factors.* |
|  | **Reduce ET:** | *Better understanding of neurobiological ‘correlates’ of mental illness could reduce ET.* | |
| Ways to contrast ET: | *Ways in which mental health professionals could contribute to contrast ET.* | | |
|  | **Refer to clinical guidelines / evidence:** | *Mental health professionals should refer to the latest clinical guidelines / latest scientific evidence in shaping their practice.* | |
|  | **Effective / communication:** | *Mental health professionals should foster good communication with clients on the significance and implications of neurobiology.* | |
|  | **Promote hope and optimism:** | *Mental health professionals should promote hope and instil a sense of optimism in clients.* | |

**IMPACT:**

| On self: | *Neurobiological explanations of psychotic illness have an impact on self / identity.* | | |
| --- | --- | --- | --- |
|  | **Illness integration:** | *Mental illness viewed as something part of self (e.g. my brain, my genes, my biology), and thus integrated within the identity. Mostly positive consequences.* | |
|  |  | **Promote resilience:** | *Illness integration promotes resilience (e.g. learning how to accept symptoms, how to deal with symptoms, instead of aiming for full remission).* |
|  | **Undermine agency and recovery:** | *Neurobiological explanations of psychotic illness may undermine the sense of agency of clients, and their perceived potential for recovery.* | |
| On families: | *Neurobiological explanations of psychotic illness impact on families of (young) people.* | | |
|  | **Risk of family conflict / distress:** | *Neurobiological explanations of psychotic illness may generate conflicts and distress within the family (blame, guilt, and distress).* | |
|  | **Risk of paternalistic role:** | *Within a biomedical model of psychotic illness, families may tend to ‘control’ a young person with psychosis, and assume a paternalistic role.* | |
| On life choices: | *A neurobiological understanding of psychotic illness may impact on individual’s life choices, in a positive way (avoid exposing oneself to risk factors, e.g. cannabis), or negative way (avoid pursuing certain life choices, e.g. education).* | | |
|  | **Reproductive choices:** | *A neurobiological understanding of psychotic illness may impact on an individual’s reproductive choices (e.g. wish not to pass this condition to one’s offspring).* | |

**STIGMA:**

| Stigma social issue, not related to etiology: | *Social stigma attached to mental illness is independent from theories about aetiology and causation. Stigma is primarily a social / cultural issues, it has nothing to do with aetiology and causation.* | | |
| --- | --- | --- | --- |
|  | **Stigma attached to diagnosis:** | *Social stigma arises from psychiatric labels / diagnoses rather than behaviour. Labels of ‘schizophrenia’ and ‘psychosis’ generate social stigma.* | |
|  | **Impact of media / cultural discourse on stigma:** | *Media have a great impact on how stereotypes on mental illness are formed, and social stigma reproduced.* | |
| Neurobiology: | *Impact of neurobiological models of psychotic illness on social stigma and labelling.* | | |
|  | **Stigmatising:** | *Neurobiological models increase social stigma.* | |
|  |  | **Broken brain model:** | *The ‘broken brain’ model creates a category of ‘different / diverse’ individuals.* |
|  | **De-stigmatising:** | *Neurobiological models decrease social stigma.* | |
|  |  | **Illness removes responsibility:** | *Psychotic individuals are not ‘responsible’ for their illness / condition, thus not to blame.* |
|  |  | **Labels may be useful in practice** | *Diagnostic categories / labels may be useful for mental health professionals to direct clinical practice.* |

**CLINICAL TRANSLATION:**

| Potential benefits: | *Translating findings from neuroscience and genomics into clinical care may produce potential benefits.* | | |
| --- | --- | --- | --- |
|  | **Prevention:** | *Neuroscience and molecular genomics may positively impact on prevention of psychosis and schizophrenia (e.g. by identifying at-risk populations).* | |
|  | **More targeted referrals / treatments:** | *Neuroscience and molecular genomics will produce novel and better treatments / medications for psychosis and schizophrenia.* | |
| Potential harms: | *Translating findings from neuroscience and genomics into clinical care may result in harm for (young) clinical populations.* | | |
|  | **More invasive measures:** | *Diagnostic measures based on neuroscience and genomics may be more ‘invasive’ than clinical interview / assessment.* | |
|  |  | **Risks for privacy & confidentiality:** | *Using genomic / neuroscience in mental health may pose threats to clients’ privacy and confidentiality.* |
|  | **How to use that information? / no viable options:** | *Once information on genomic risk / biology of the brain is acquired, how to use that information in a clinically useful way? There may be no viable options.* | |
|  |  | **Risk of labelling:** | *There is a risk that clinical translation will only result in additional labelling of clients.* |
|  | **Risk of hopelessness/ disengagement:** | *Clinical measures based on neurobiology may perpetuate a sense of hopelessness and inevitability of psychotic illness. This could result in clients disengaging from services.* | |

**EFFECTS OF NOVEL DIAGNOSTIC TOOLS:**

| On client’s self: | *Novel diagnostic tools based on neuroscience and genomics may have an impact on client’s self /identity.* | | |
| --- | --- | --- | --- |
|  | **Depends on how information is communicated:** | *Impact on the identity of (young) clients will depend on how neurobiological information is communicated, and how it is used in the therapeutic relationship.* | |
|  |  | **Potentially harmful:** | *There is a potential for harming clients’ conception of self / identity if there is no appropriate communication.* |
|  |  | **Beneficial if effective communication:** | *Information on neurobiology may be beneficial to clients if appropriate communication strategies are in place.* |
| On clinician-patient relationship: | *Novel diagnostic tools based on neuroscience and genomics may have an impact on clinician – patient relationship.* | | |
|  | **Potential conflict on therapy:** | *Using diagnostic tools based on neurobiology could generate tensions between professionals and clients on therapeutic options. Risk of ‘moral coercion’ towards therapeutic options.* | |
|  | **Psychosis risk communication:** | *Moral challenges of communicating psychosis risk to help-seeking or asymptomatic individuals.* | |
|  |  | **Risk is not inevitability:** | *Mental health professionals should communicate that psychosis risk is not inevitability to develop psychotic illness.* |
|  |  | **Psychosis very common in general population:** | *Mental health professionals should communicate that psychosis is very common in the general population, and is not equal to psychotic illness.* |
| On practice: | *Novel diagnostic tools based on neuroscience and genomics may have an impact on clinical practice.* | | |
|  | **Risk of medicalisation:** | *There is risk to medicalise mental and behavioural difficulties.* | |
|  | **Risk of over-diagnosis:** | *Using novel diagnostic tools based on neurobiology could result in over-diagnosing people.* | |
|  |  | **Over-diagnosis is already happening:** | *Over-diagnosis of psychotic illness is already happening / is part of the history of psychiatry.* |
|  |  | **Neurobiology could reduce misdiagnosis:** | *Diagnostic tools based on neurobiology could REDUCE misdiagnosis / wrong diagnosis of psychotic illness.* |
| On services: | *Novel diagnostic tools based on neuroscience and genomics may have an impact on how mental health services work.* | | |
|  | **Possible professional conflict:** | *Different professionals will probably react in very different ways, thus possibly generating moral / ethical conflicts.* | |
|  | **Clinicians’ response:** | *Reaction of mental health professionals to the introduction of neuroscience / genomic-based diagnosis / treatment.* | |
|  |  | **Scepticism:** | *Most mental healthcare providers may be sceptical of the clinical utility of translational efforts.* |
|  |  | **Based on background:** | *Clinician’s reaction may be affected by their professional background.* |

**GENETIC TESTING:**

| Impact: | *(Hypothetical) genetic testing for predisposition to psychosis or schizophrenia may affect individuals.* | | |
| --- | --- | --- | --- |
|  | **On at-risk individuals:** | *Impact of GT on individuals already identified as being at-risk through clinical assessment.* | |
|  |  | **Affect views on treatability:** | *GT could affect client’s views on treatability of psychotic illness (e.g. untreatable / fatalistic / useless talking therapies).* |
|  |  | **Remove control over illness:** | *GT could instil a sense of lack of control over the illness / undermine potential for recovery.* |
|  | **On reproductive choices:** | *GT may impact on an individual’s reproductive choices (e.g. wish not to pass the condition to one’s offspring).* | |
|  | **On families:** | *GT may impact on family relationship.* | |
|  |  | **Blame and guilt:** | *GT may generate feelings of blame and guilt for ‘passing on the illness / being responsible for someone else’s psychosis.’* |
| Harms: | *(Hypothetical) genetic testing for predisposition to psychosis or schizophrenia may generate potential harms.* | | |
|  | **Risk of disengagement from services:** | *Risk that clients disengage from mental health services after receiving information on genetic predisposition to psychotic illness.* | |
|  | **Risk of hopelessness / determinism:** | *Risk to instil a sense of hopelessness towards recovery in clients / sense of determinism in developing psychotic illness.* | |
|  | **Risk of discrimination: Insurance / jobs:** | *Risk of discrimination that could derive from holding genetic information on predisposition to psychosis and schizophrenia (e.g. insurance, job applications, benefit applications).* | |
|  |  | **Discrimination already present:** | *Discrimination towards people with mental illness is already present, and it may be exacerbated by GT.* |
|  |  | **Risk of information leak (always on record)** | *Risk of information leak with regard to predisposition to psychotic illness / risk that such information may always stay on a ‘person’s record’.* |
| Benefits | *(Hypothetical) genetic testing for predisposition to psychosis or schizophrenia may generate potential benefits.* | | |
|  | **Could have CU where there is a long family history of MI:** | *GT for predisposition to psychosis or schizophrenia may be beneficial and support mental health professionals where there is long family history of mental illness. Clinical benefits could be gained through prevention and early intervention.* | |
